# Isolated gastric recurrence from ovarian carcinoma: A case report

**DOI:** 10.3892/ol.2015.2887

**Published:** 2015-01-19

**Authors:** QIAN LIU, QIAN-QIAN YU, HAO WU, ZHI-HONG ZHANG, REN-HUA GUO

**Affiliations:** 1 Department of Oncology, Huaiyin Hospital of Huai’an City, Huai’an, Jiangsu 223300, P.R. China; 2 Department of Oncology, The First Affiliated Hospital of Nanjing Medical University, Nanjing, Jiangsu 210029, P.R. China; 3 Department of Pathology, The First Affiliated Hospital of Nanjing Medical University, Nanjing, Jiangsu 210029, P.R. China

**Keywords:** gastric metastasis, ovarian carcinoma, International Federation of Gynecology and Obstetrics cancer staging, ^18^F-fluorodeoxyglucose positron emission tomography/computed tomography, recurrent disease, serum carbohydrate antigen-125

## Abstract

Although ovarian metastasis secondary to gastric cancer (Krukenberg tumor) has been extensively described in the literature, gastric metastasis from ovarian carcinoma is rare. The present case report describes a patient with gastric metastasis from ovarian carcinoma. A 51-year-old female with previously treated ovarian carcinoma of stage III according to the International Federation of Gynecology and Obstetrics was admitted to the Department of Oncology, First Affiliated Hospital of Nanjing Medical University (Nanjing, China) with high serum carbohydrate antigen-125 levels. Endoscopic ultrasound and ^18^F-fluorodeoxyglucose positron emission tomography/computed tomography scanning revealed a lesion in the stomach with the typical appearance of a gastrointestinal stromal tumor. The histopathological examination revealed infiltration of the resected specimens by metastatic serous adenocarcinoma and a comparison with the previously resected ovarian specimen confirmed disease recurrence. Although isolated gastric recurrence from ovarian carcinoma is rare, when a patient has a history of ovarian carcinoma (particularly with a high CA-125 level) and when the imaging results show a mass in the stomach wall, metastasis from ovarian carcinoma should be considered.

## Introduction

Ovarian metastasis secondary to gastric cancer (Krukenberg tumor) has been extensively described in the literature (1,2); however, gastric metastasis from ovarian carcinoma has rarely been reported. An isolated parenchymal gastric metastasis from ovarian carcinoma without any other sites of recurrence is an extremely rare event, with only two cases reported in China (3,4). Isolated gastric metastasis in the absence of peritoneal seeding suggests hematogenous spread of the tumor (3,5). The reports on this route of metastasis are limited and the majority of these cases were diagnosed in asymptomatic patients during follow-up by elevated serum carbohydrate antigen (CA)-125 levels, or by images revealing the presence of gastric tumors suggestive of gastrointestinal stromal tumors (3,5–10). The present report describes a case of an isolated gastric recurrence from ovarian carcinoma in a 51-year-old asymptomatic patient. The diagnosis was histologically confirmed following surgical resection. Written informed consent was obtained from the patient.

## Case report

**Figure 1:**
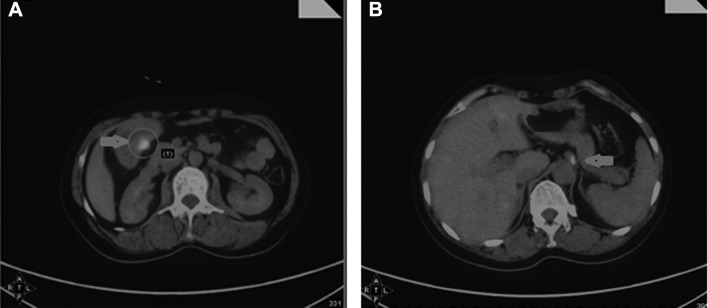
^18^F-fluorodeoxyglucose positron emission tomography/computed tomography showing (A) a hypermetabolic lesion in the gastric antrum (arrow) and (B) a high-uptake lymph node behind the pancreas (arrow).

**Figure 2:**
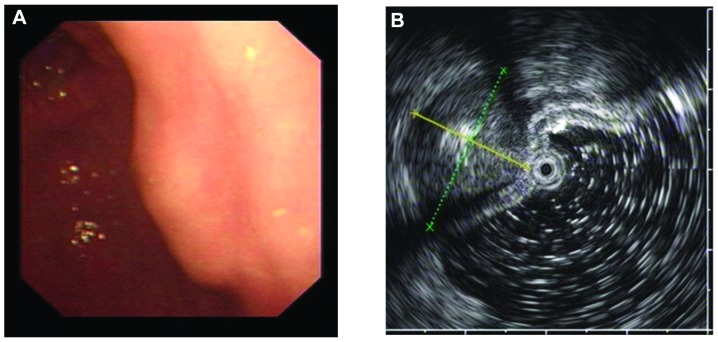
(A) Endoscopic view of the protruding tumor in the gastric antrum. (B) Endoscopic ultrasound image of a 1.5×2.0-cm hypoechoic mass emanating from the muscularis propria with the typical appearance of gastrointestinal stromal tumor.

**Figure 3:**
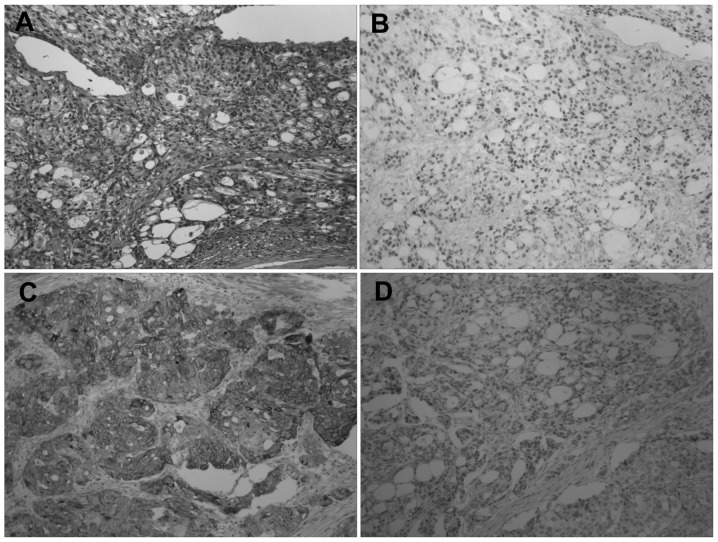
Pathological examination of the neoplasm (magnification, ×100). (A) Microscopically, the tumor is composed of irregular sheets of cells with high-grade nuclear atypia (hematoxylin and eosin stain). Immunohistochemically, the tumor cells are immunoreactive for (B) estrogen receptor, (C) cytokeratin 7 and (D) Wilms’ tumor-1.

**Figure 4:**
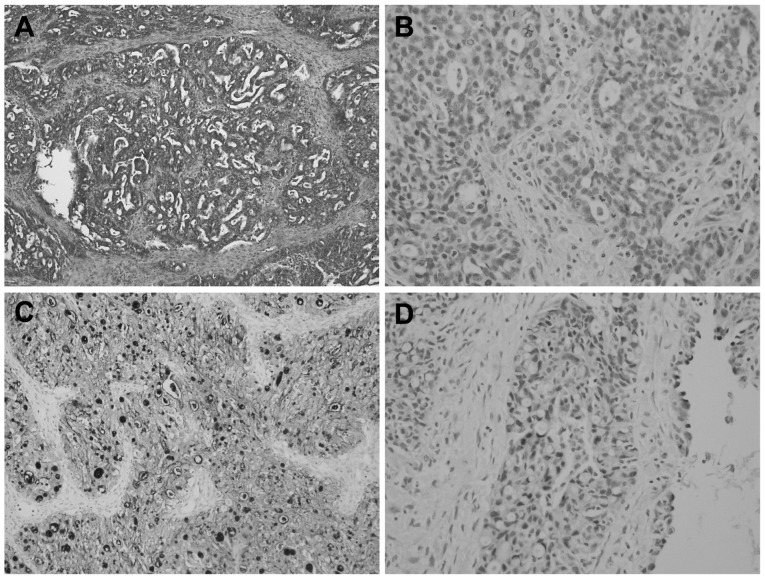
(A) Hematoxylin and eosin staining of ovarian cancer (magnification, ×100). Immunohistochemically, the tumor cells are immunoreactive for (B) estrogen receptor (magnification, ×200), (C) CA-125 (magnification, ×40) and (D) Wilms’ tumor-1 (magnification, ×400).

In April, 2012, a 51-year-old female presented to the Department of Gynecology of the First Affiliated Hospital of Nanjing Medical University (Nanjing, China) complaining of lower abdominal pain of two months in duration. A computed tomography (CT) scan of the abdomen and pelvis showed bilateral adnexal complex masses. The patient subsequently underwent a total abdominal hysterectomy, bilateral salpingo-oophorectomy with pelvic lymph node dissection and omentectomy for stage III ovarian adenocarcinoma (International Federation of Gynecology and Obstetrics). The surgery was followed by four cycles of adjuvant chemotherapy with paclitaxel and cisplatin. The patient was in good condition and achieved complete clinical response in June, 2012. In December, 2013, the patient was admitted to the Department of Oncology, First Affiliated Hospital of Nanjing Medical University due to significantly elevated serum CA-125 levels (up to 96.05 U/ml; normal, <35 U/ml), which is an important tumor marker. Other tumor markers were within the normal range. The patient had no abdominal discomfort, hematemesis, melena, weight loss or any other clinical manifestations. Scanning with ^18^F-fluorodeoxyglucose positron emission tomography(^18^F-FDG PET)/CT ruled out recurrent ovarian carcinoma, which was suspected due to the high CA-125 levels. The ^18^F-FDG PET/CT images revealed a high-uptake lesion in the antrum of the stomach (Fig. 1A) and a high-uptake lymph node behind the pancreas (Fig. 1B). During the subsequent endoscopic examination, a 1.5×2.0-cm submucosal mass covered with normal gastric mucosa was identified in the gastric antrum (Fig. 2A). Due to the location of the lesion, the patient was referred for endoscopic ultrasound examination, which revealed a hypoechoic mass emanating from the muscularis propria, with the typical appearance of a gastrointestinal stromal tumor (Fig. 2B). The patient subsequently underwent surgical resection of the gastric lesion and the intumescent lymph nodes. The intraoperative findings included a 2.5×2×2-cm isolated extrinsic mass on the wall of the gastric antrum and a 1.0×0.8-cm intumescent lymph node close to the lesser curvature of the stomach. The histopathological examination confirmed ovarian cancer relapse involving the gastric wall without mucosal involvement, although a lymph node was involved. Immunostaining for progesterone receptor, estrogen receptor, cytokeratin 7 (CK7) and Wilms’ tumor-1 (WT1) was positive; while immunostaining for CK20 was negative (Fig. 3). These findings were consistent with the primary ovarian cancer (Fig. 4). The patient was discharged on the 10th day after surgery. At the time of writing, the patient had received another two cycles of adjuvant chemotherapy, and the CA-125 level had decreased to the normal range.

## Discussion

Gastric metastasis is a rare occurrence. A previous study on 1,010 patients with malignant tumors reported 17 cases (1.7%) of metastasis to the stomach (11). The most prevalent primary sites are the lung, breast, skin (melanoma) and esophagus (12). Ovarian tumor metastasis to the stomach is uncommon. To the best of our knowledge, only 10 reports have been published to date (3–10,13,14). The common metastatic sites throughout the abdominal cavity are the intestinal omentum, mesentery and serosa (6). Another study including 50 patients identified 67 distant metastatic sites: Liver, 21; pleura, 11; lung, eight; central nervous system and skin, seven each; extra-abdominal lymph nodes and spleen, five each; bone, two; and breast, one (15). Although the intraperitoneal route of dissemination is considered to be the most common, ovarian cancer may also metastatize through the lymphatic channels and the hematogenous route (16). We consider that the latter route may account for the ovarian cancer metastasis in our case.

Immunohistochemistry is crucial for distinguishing primary ovarian adenocarcinoma from metastatic adenocarcinomas, particularly those of gastrointestinal origin (17). WT1 is expressed at a high frequency in patients with epithelial ovarian cancer, and it is significant in determining whether a serous carcinoma is primary or metastatic (18). CK7 and CK20 are two of the most commonly used tumor-detecting diagnostic tools in surgical pathology. CK7 shows diffuse and strong staining in all serous ovarian tumors, but the majority of metastatic gastrointestinal carcinomas are negative for CK7 (19). CK20 has been found to be negative in almost all ovarian tumors (20). When attempting to differentiate between primary gastric cancer and metastasis from ovarian cancer, it should be noted that a monoclonal antibody panel for WT1, CK7 and CK20 may facilitate this discrimination.

Recurrent ovarian carcinoma remains a therapeutic issue for physicians. To date, there is no consensus regarding optimal treatment strategies. Secondary cytoreductive surgery may be considered for patients who present with recurrence after a long disease-free period (≥6 months) (21). A recent meta-analysis suggested that patients with recurrent disease who undergo complete cytoreduction achieve prolonged survival (22).

The clinical manifestations of gastric metastasis from ovarian carcinoma are diverse and non-specific. Including the present case, five cases were asymptomatic (3,4,7,8), one presented with epigastric pain and fullness (6), three presented with gastrointestinal hemorrhage (9,10,13), and one presented with belching and reflux (5). Therefore, when a patient has a history of ovarian carcinoma, particularly with high CA-125 levels, and the imaging results reveal a mass in the gastric wall, metastasis from ovarian carcinoma should be considered. Imaging with ^18^F-FDG PET/CT and endoscopic ultrasound may be useful for correctly diagnosing gastric lesions and should be considered in all cases if available.

Although metastasis of ovarian tumor to the stomach is rare, clinicians should consider that, in patients with a history of ovarian cancer, gastric lesions may be secondary metastases from ovarian cancer.
